# Surgical correction of total anomalous pulmonary venous return in an adult patient

**DOI:** 10.1186/s13019-022-01990-9

**Published:** 2022-09-19

**Authors:** Yohan Bae, Woo Sung Jang, Kyungsub Song

**Affiliations:** Department of Thoracic and Cardiovascular Surgery, Dongsan Medical Center, Keimyung University School of Medicine, 1035, Dalgubeol-daero, Dalseo-gu, Daegu, 42601 Republic of Korea

**Keywords:** TAPVR, Adult congenital heart surgery, Vascular anomalies, Complex congenital heart disease

## Abstract

**Background:**

Total anomalous pulmonary venous return (TAPVR) is rare congenital heart disease. Most TAPVRs require surgical corrections in the neonatal period and survival to adulthood without surgical correction is extremely rare. Most untreated patients with large atrial septal defects and no pulmonary venous obstruction have pulmonary vascular damage from pulmonary over circulation.

**Case presentation:**

44-year-old TAPVR patient admitted to our medical center. A snowman-shaped heart, including cardiomegaly and an increase in pulmonary blood flow, was seen in the chest X-ray. A large-sized (around 3 cm) atrial septal defect with dilated right atrium, right ventricle, and pulmonary artery was detected on echocardiography. Heart computed tomography was performed for further evaluation, and supra-cardiac type TAPVR without any obstructive lesion was identified.

**Conclusions:**

TAPVR in an adult patient is extremely rare, and this patient was treated successfully with surgical correction and is doing well. A sinus rhythm and mild mitral valve regurgitation have remained during 2.5 years of outpatient follow-up.

A 44-year-old man was admitted for acute onset palpitations and dyspnea. On physical examination, clubbed fingers were observed. The patient’s oxygen saturation was 89% in room air, and atrial tachycardia was seen on electrocardiography. Blood chemistry evaluation showed elevated liver enzymes (aminotransferase/alanine aminotransferase: 2060/1649 U/L, total bilirubin/ direct bilirubin 4.41/0.84 mg/dL) and amino-terminal pro-brain natriuretic peptide (NT-proBNP) levels up to 6789 pg/mL. A snowman-shaped heart, including cardiomegaly and an increase in pulmonary vasculature, was seen in the chest X-ray (Fig. 1). Cardiomegaly on chest X-ray was detected 23 years ago and noted in his past medical history. However, there was no follow-up.

**Fig. 1:**
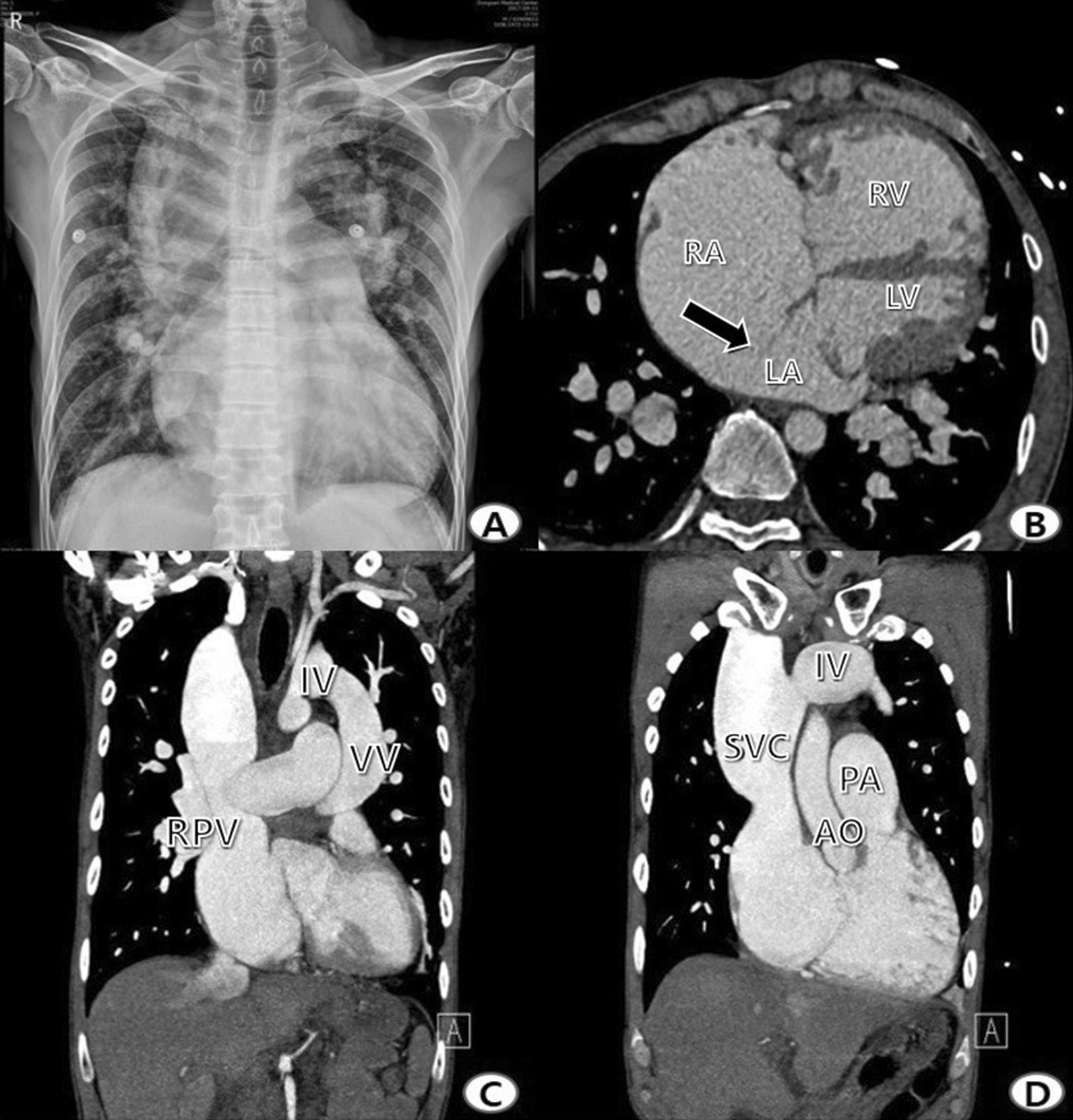
Preoperative chest X-ray and computed tomography (CT). **A** Preoperative chest X-ray. **B** Axial view on CT shows an enlargement of the RA and RV, secundum-type ASD (arrow), and left dislocation of the LA and LV. **C**, **D** Coronal view on CT shows that the IV is connected to the VV and drains to the SVC. RA, right atrium; RV, right ventricle; ASD, atrial septal defect; LA, left atrium; LV, left ventricle; IV, innominate vein; VV, vertical vein; RPV, right pulmonary vein; SVC, superior vena cava; PA, pulmonary artery; AO, aorta

A large-sized (around 3 cm) atrial septal defect (ASD) with dilated right atrium, right ventricle, and pulmonary artery was detected on echocardiography. In addition, a D-shaped left ventricle (LV) and severe mitral valve regurgitation were also detected. Sinus rhythm recovered after digitalization and diuretic administration. The mitral valve regurgitation was improved to a mild degree on echocardiography, and the liver enzymes were also normalized. Cardiac computed tomography was performed for further evaluation, and supra-cardiac type total anomalous pulmonary venous return (TAPVR) without any obstructive lesion was identified. The pulmonary venous drainage course, including the superior vena cava was severely dilated. However, the left atrium and left ventricle sizes were adequate (Fig. 1). The pulmonary arterial pressure was 43/22 mmHg (Mean: 29 mmHg) on right heart catheterization (Table 1).

**Table 1:** Preoperative cardiac catheterization data

Site	O2 saturation (%)	Pressure systolic/diastolic pressure (mean), mmHg
Superior vena cava	90.7	
Right atrium	85.4	12/4 (7)
Right ventricle	88.7	43/8 (20)
Pulmonary artery	88.7	43/22 (29)
Left atrium	89.6	12/5 (7)
Aorta	90	100/60 (73)

We performed a direct anastomosis of the pulmonary venous confluence to the left atrium and ASD patch closure by the transverse sinus approach without total circulatory arrest. The vertical vein was ligated. The cardiopulmonary bypass (CPB) and aortic cross-clamp times were 147 and 61 min, respectively. We also checked the mitral valve morphology. The mitral valve showed diffuse leaflet thickening at the anterior and posterior valves. However, the coaptation margin of the valve leaflet was good. Thus, we did not perform mitral valve surgery. The patient weaned off CPB easily. On postoperative TEE, No turbulence was observed across the anastomosis, and the mitral valve regurgitation was mild. The patient was extubated on postoperative day (POD) 0 and transferred to the general ward on POD 1. He was discharged from hospital on POD 8 without any symptoms. The patient is doing well. The cardiac rhythm remains in sinus rhythm and transthoracic echocardiography show mild mitral valve regurgitation after 2.5 years of operation (Fig. 2).

**Fig. 2:**
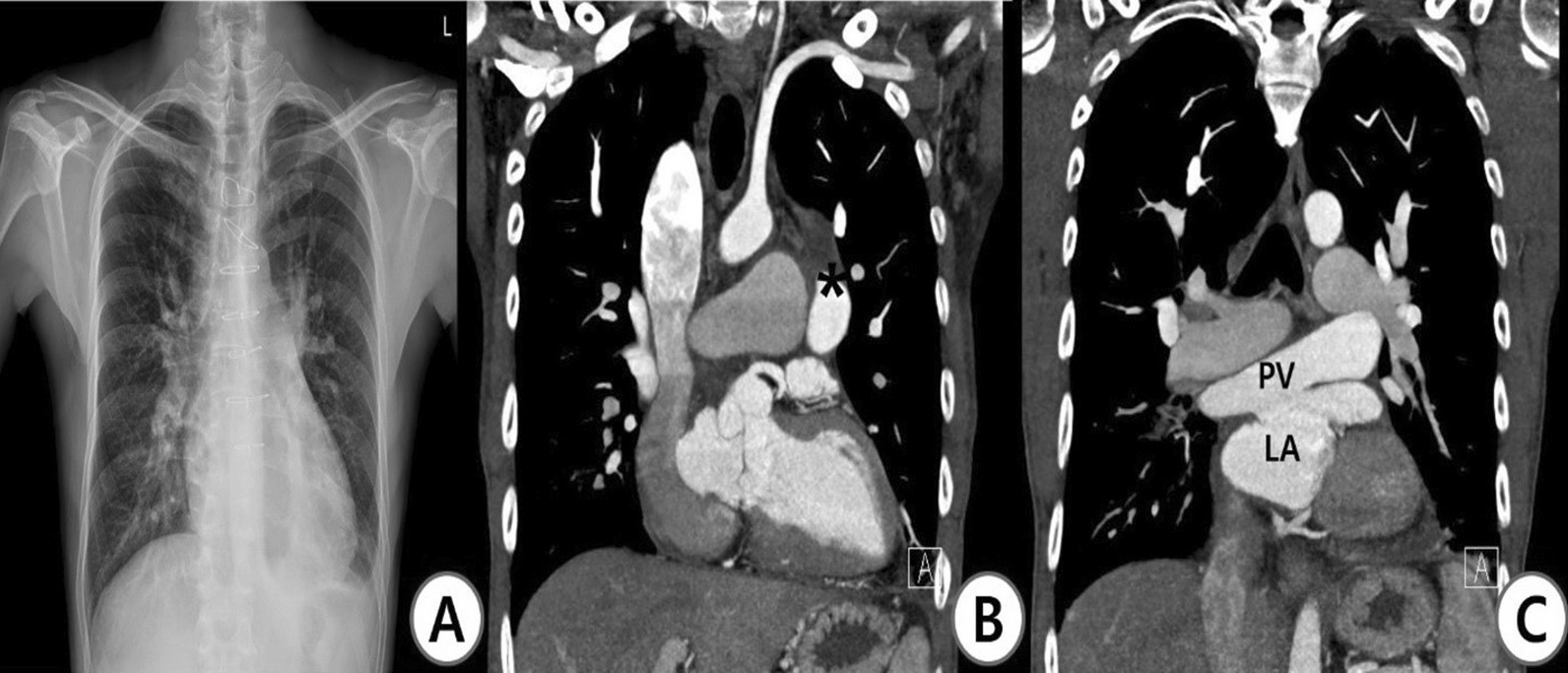
Postoperative computed tomography (CT). **A** Chest X-ray at discharge. **B**, **C** Coronal view on chest CT at 2.5 years after surgery. The PV was connected to the LA. PV, pulmonary vein; LA, left atrium. *ligated vertical vein

## Discussion

TAPVR is a rare anomaly accounting for only 1.5% to 2.2% of congenital heart disease [1]. TAPVR is a cardiac malformation in which all pulmonary veins on either side are not connected to the left atrium, but directly to the somatic vein or the right atrium. Almost all patients with TAPVR present with symptoms of heart failure in the neonatal period, which require early surgical correction. It is unusual to encounter a adult patient with TAPVR due to low survival rate in untreated cases.

According to Talwar et al. [2] in group of 27 patients who underwent surgery for TAPVR at the age of 15–48 years, Unobstructed pulmonary venous drainage, a larger inter-atrial communication, maintenance of vascular resistance and pulmonary pressure closer to normal limits was important factors for survival in unoperated patients with TAPVR. Like this authors believes, Our patient also had mild pulmonary hypertension, supracardiac type TAPVR with non restrictive ASD and absence of pulmonary obstruction. This condition is major factors for long term survival in adult TAPVR patients.

If patients not had pulmonary vascular disease, most patients are able to complete repair. But with severe pulmonary artery hypertension, Right heart decompression such as small interatrial communication, partial closure of the septal defect and unidirectional valve patch, should be performed [3]. All these techniques were left to permit right-to-left shunting during pulmonary hypertensive crisis. Alternate surgical option had been reported by India group about test protocol for selective ligation of vertical vein [4]. A patent vertical vein functions like an atrial septal defect, when too small noncompliant atrium to accommodate blood flow after surgical repair. But this technique was potential hazard to left-to-right shunt. Especially in pulmonary hypertensive crisis with pulmonary arteriolar constriction, Left-to-right shunt results in lower preload of left ventricle which exacerbates cardiac output reduction.

There are few adult TAPVR patients worldwide. The cases reported previously showed a favorable prognosis in the postoperative phase. However, approximately 10% to 15% of the patients have evidence of late pulmonary vein obstruction, which tends to be recurrent and progressive [5]. In the three years of follow-up after surgery, this patient did not show any complications, including pulmonary venous obstruction or mitral valve regurgitation. However, long-term surveillance and monitoring are required.

## Data Availability

Not applicable.

## Abbreviations

NT-proBNP: NT-amino-terminal pro-brain natriuretic peptide
ASD: Atrial septal defect
LV: Left ventricle
TAPVR: Total anomalous pulmonary vein return; CPB: Cardiopulmonary bypass
POD: Postoperative day
